# Novel Bioactive and Antibacterial Acrylic Bone Cement Nanocomposites Modified with Graphene Oxide and Chitosan

**DOI:** 10.3390/ijms20122938

**Published:** 2019-06-15

**Authors:** Mayra Eliana Valencia Zapata, José Herminsul Mina Hernandez, Carlos David Grande Tovar, Carlos Humberto Valencia Llano, José Alfredo Diaz Escobar, Blanca Vázquez-Lasa, Julio San Román, Luis Rojo

**Affiliations:** 1 Grupo de Materiales Compuestos, Escuela de Ingeniería de Materiales, Universidad del Valle, Calle 13 # 100-00, Cali 76001, Colombia; valencia.mayra@correounivalle.edu.co (M.E.V.Z.); jose.mina@correounivalle.edu.co (J.H.M.H.); 2 Grupo de Investigación de Fotoquímica y Fotobiología, Universidad del Atlántico, Carrera 30 Número 8-49, Puerto Colombia 081008, Colombia; 3 Grupo Biomateriales Dentales, Escuela de Odontología, Universidad del Valle, Calle 13 No. 100-00, Cali 76001, Colombia; carlos.humberto.valencia@correounivalle.edu.co; 4 Departamento de Ciencias Básicas, Institución Universitaria Antonio José Camacho, Avenida 6N # 28N – 102, Cali 76001, Colombia; afro77777@gmail.com; 5 Instituto de Ciencia y Tecnología de Polímeros, ICTP-CSIC, C/Juan de la Cierva 3, 28006 Madrid, Spain; bvazquez@ictp.csic.es (B.V.-L.); jsroman@ictp.csic.es (J.S.R.); 6 Consorcio Centro de Investigación Biomedica en red, CIBER-BBN, 28029 Madrid, Spain

**Keywords:** acrylic bone cement, chitosan, graphene oxide, nanocomposite

## Abstract

Acrylic bone cements (ABCs) have played a key role in orthopedic surgery mainly in arthroplasties, but their use is increasingly extending to other applications, such as remodeling of cancerous bones, cranioplasties, and vertebroplasties. However, these materials present some limitations related to their inert behavior and the risk of infection after implantation, which leads to a lack of attachment and makes necessary new surgical interventions. In this research, the physicochemical, thermal, mechanical, and biological properties of ABCs modified with chitosan (CS) and graphene oxide (GO) were studied. Fourier transform infrared (FTIR) spectroscopy, proton nuclear magnetic resonance (^1^H-NMR) scanning electron microscopy (SEM), Raman mapping, thermogravimetric analysis (TGA), differential scanning calorimetry (DSC), compression resistance, mechanical dynamic analysis (DMA), hydrolytic degradation, cell viability, alkaline phosphatase (ALP) activity with human osteoblasts (HOb), and antibacterial activity against Gram-negative bacteria *Escherichia coli* were used to characterize the ABCs. The results revealed good dispersion of GO nanosheets in the ABCs. GO provided an increase in antibacterial activity, roughness, and flexural behavior, while CS generated porosity, increased the rate of degradation, and decreased compression properties. All ABCs were not cytotoxic and support good cell viability of HOb. The novel formulation of ABCs containing GO and CS simultaneously, increased the thermal stability, flexural modulus, antibacterial behavior, and osteogenic activity, which gives it a high potential for its uses in orthopedic applications.

## 1. Introduction

Due to the increase in the aging population and the increase of sport-related trauma, the number of patients undergoing joint replacement surgery is increasing, particularly in young people [1,2]. Most prostheses are being fixed using acrylic bone cements (ABCs) of poly(methyl methacrylate) (PMMA), which acts as a glue between the implant and the bone and is characterized as an inert material [3]. Most commercial ABCs currently available consist of two components, a solid phase composed mainly of a polymer based on methyl methacrylate (MMA) and a liquid phase based on MMA [4], which are mixed to produce the cement, due to the free radical polymerization reaction of the monomer present [1].

The main advantages of cemented prostheses are their excellent primary fixation, the uniform distribution of the load between the implant and the bone, and the fact that the technique allows rapid recovery of the patient. However, the main disadvantage is that about 10% of patients may require revision in less than ten years after implantation [5]. 

The observed aseptic loosening that appears over time is attributed to causes such as the lack of secondary fixation, mechanical failure of the cement, and the initial formation of fibrous tissue between the cement and the bone. This is mainly due to bone necrosis induced by the heat released during the polymerization reaction, and osteolysis caused by the foreign body reaction induced by wear particles which could come from the bone cement. The toxicity of the liquid monomer of cement is also another disadvantage. Septic loosening, produced by bacterial infections, is a common complication after a total joint replacement with infection rates up to 3% [6]. In spite of the above, ABCs still have a long way to go and a future ahead, since no material has played such an important role and has been in widespread use in orthopedics in the last 60 years [3,4]. 

With the aim of improving the limitations, there are studies in which ABCs imparted bioactivity through the addition of inorganic particles. These approaches led to strengthening of the cement–bone interface but had repercussions on losses in the mechanical properties of cement, which also compromises the longevity of the prosthesis. On the other hand, to prevent the formation of biofilm caused by bacteria, work has been done to control the release of antibiotics by including biodegradable polymers [7], which has achieved relatively good short-term results. However, despite the multiple strategies proposed for durable antibacterial ACBs [8,9], the appearance of resistant bacteria and the loss of mechanical properties over time persist and this makes it is necessary to investigate new materials capable of combating these drawbacks.

Chitosan is a polymer of great interest for biomedical applications and implants [10], because of its intrinsic properties, such as biocompatibility, antibacterial activity [11], bioactivity [12], controllable degradation rate [13], and osteoconductive and osteoinductive activities [14]. Additionally, chitosan is obtained from renewable sources that are currently extending its applications. CS has been combined with a variety of polymeric biomaterials such as alginate, hyaluronic acid, PMMA or poly-L-lactic acid (PLLA), and inorganic bioactive compounds, like hydroxyapatite or calcium phosphate, and growth factors, for potential application in orthopedics as bone graft substitutes, intervertebral discs, and bone and cartilage tissue engineering [14,15]. Meanwhile, the use of graphene oxide (GO) nanoparticles in biomedical applications has become a hot topic of research. GO can provide mechanical reinforcement to biomaterials like chitosan [11], polycaprolactone [16] and polylactic acid [17] among others. GO possesses intrinsic antibacterial effects against Gram-positive bacteria like *Staphylococcus aureus* and Gram-negative bacteria like *Escherichia coli* that makes it a potential agent in the treatment of resistant bacterial infections [18]. Moreover, due to its low cytotoxicity, GO can be a promising candidate in orthopedic applications for regeneration of bone tissue [19], in bone cements [20], and in cartilage regeneration [21]. 

This work studied the unexplored effect of combined CS and GO on ABC nanocomposites and their potential to be used as antibacterial bioactive cement in orthopedic applications. The effect of the addition of each component (15% CS and 0.3% GO) was studied together and separately. The ABCs formulations were characterized by analyzing their physicochemical, thermal, and mechanical properties. Likewise, the swelling and degradation behavior of the modified cements were evaluated in vitro in simulated body conditions, their antibacterial activity assessed against Gram-negative bacteria and their biological performance studied in human osteoblast cell cultures.

## 2. Results and Discussion

### 2.1. Characterization of Graphene Oxide

**Figure 1:**
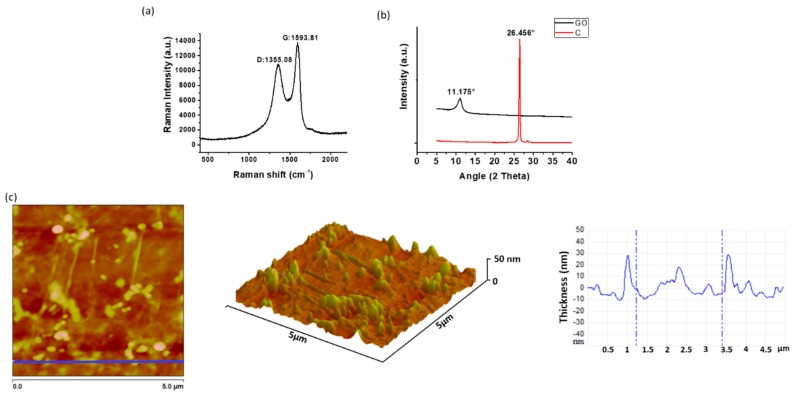
Characterization of GO by (**a**) Raman, (**b**) XRD (C is graphite), and (**c**) atomic force microscopy (AFM). Analysis of GO performed on tapping mode showing topography and thickness.

Figure 1 shows the results of the GO characterization. The Raman spectrum (Figure 1a) exhibits the characteristic bands of the GO. The G band is attributed to the stretching of the bond of the carbon pairs in sp^2^ hybridization in the graphite, and the D band is assigned to the breathing mode of the sp^2^ carbon rings and indicates the existence of defects in the graphite network due to the presence of oxygen-rich functional groups [22]. X-ray diffraction (XRD) patterns for graphite and GO (Figure 1b) show that, in graphite, the peak corresponding to the reflection of the plane (002) appears at an angle 2θ = 26.46°, while for the GO that peak appears at 2θ = 10.77° and corresponds to the reflection of the plane (001) [23,24]. Applying Bragg’s law to these results, it is obtained that the interlaminar distance increases from 3.36 Å for the graphite to 7.953 Å for the GO due to the presence of the oxygen-rich functional groups in the latter [25]. The increase in the interplanar distance facilitates the dispersion of the GO sheets in different media. The values obtained in this investigation approach to those reported by other authors [11]. The GO nanosheets used in this study had an average size of 400 nm with a polydispersity (PDI) of 0.055 measured by dynamic light scattering (DLS) (Figure S1).

The morphology of the GO sheets was studied by atomic force microscopy (AFM), which is shown in Figure 1c. It can be observed that the thickness of the nanosheets is lower than 30 nm, confirming that there is little overlapping of GO sheets [26].

### 2.2. Characterization of ABC

#### 2.2.1. Chemical Characterization

• FTIR Spectroscopy

**Figure 2:**
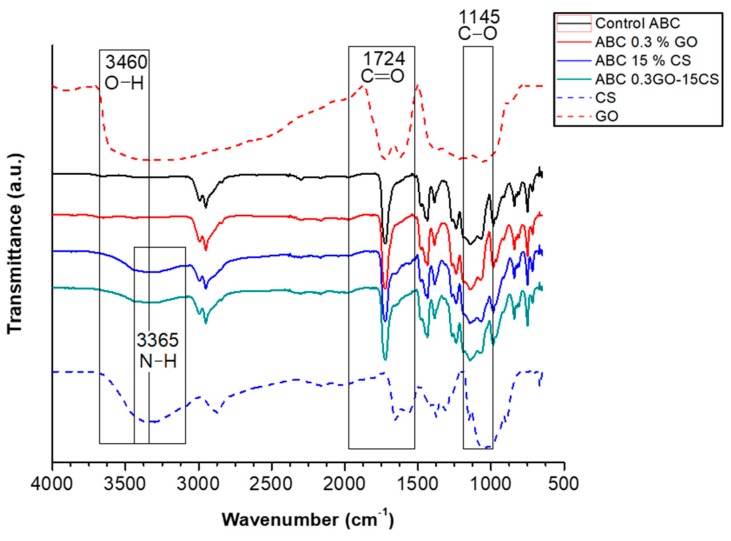
ATR-FTIR spectra of ABCs, CS, and GO. Control ABC corresponds to ABC without fillers.

Figure 2 shows the Fourier-transform infrared (FTIR) spectra of ABCs and those of the pure added fillers. The characteristic bands of GO [25] and CS, [27,28,29,30] were present in the ABCs spectra. In particular, the band corresponding to the amino group (3365 cm^−1^) did not vary in the position with respect to the pristine CS, which corroborates that there is no chemical interaction between the CS and the PMMA. In addition, no variations were observed in the spectra of ABCs with GO attributable to the incorporation of GO, mainly due to the low proportion of this component in the formulation and, also, to the fact that the main absorption bands of GO overlap with those of PMMA. 

• Residual Monomer

The amount of the residual monomer content of the ABCs nanocomposites obtained by proton nuclear magnetic resonance (^1^H-NMR) is shown in Table 1. The results indicate that the incorporation of each component GO or CS separately hardly increased the residual monomer content of the cements. However, when both components were added together the value moderately increased. This increasing of the residual monomer with the presence of fillers can be due to a lower diffusion capacity of the monomer through the polymerizing mass during the polymerization process, leading to more moderate heat dissipation. Other authors have reported an increase in the amount of residual monomer with the incorporation of GO [20] and CS. Despite the rise, these values are lower than those reported in other studies [20,31] and are similar to those reported by Islas-Blancas et al. [32], who affirm that they are within the range observed for commercial cement [33]. 

#### 2.2.2. Physical Characterization

• SEM

**Figure 3:**
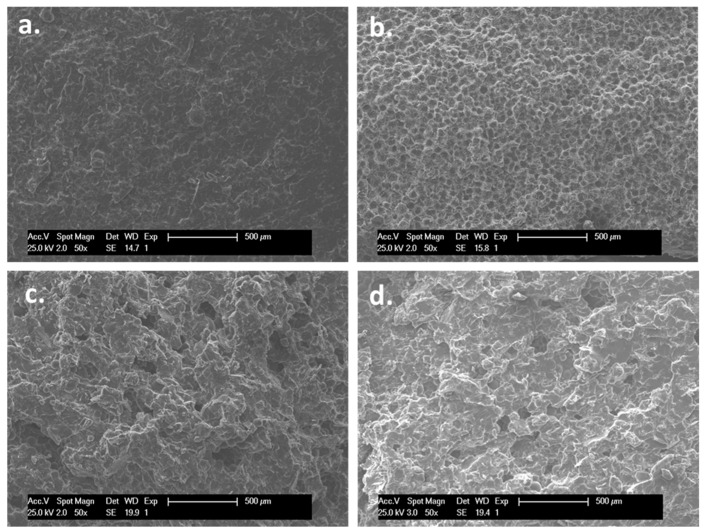
The SEM images (50×) of (**a**) control ABC, (**b**) ABC with 0.3% GO, (**c**) ABC with 15% CS, and (**d**) ABC with 0.3% GO and 15% CS samples.

According to the scanning electron microscopy (SEM) images reported in Figure 3, the morphology of the ABCs nanocomposites varies significantly with the addition of fillers. Compared with the control ABC (Figure 3a) the GO (Figure 3b) generates a rough surface, as reported by Gonalves et al. [34], which is attributed to the morphology of the nanosheets. On the other hand, the presence of CS in the formulation (Figure 3c) contributes to the porosity because the higher viscosity of the paste generated by the presence of the CS promotes the entrapment of air inside the cement during the mixing process. The formulation with both charges (CS and GO) (Figure 3d) presented a synergy in the morphology, reflecting a surface with higher roughness and porosity compared with the control.

From a biological point of view, the morphological features in terms of roughness and porosity have a major effect on the surface biocompatibility by promoting good anchoring pattern for cells. For instance, Khandaker et al. reported that an increase in the roughness of acrylic bone cements with the incorporation of polycaprolactone electrospun nanofibers generated an increase in cytocompatibility of osteoblast cells, their adhesion, proliferation, mineralization, and protein adsorption [35]. On the other hand, it has been reported that roughness plays a key role in bone contact biomaterials, such as titanium, increasing cell growth and migration and expression of proteins in the extracellular matrix [36]. 

• Raman Mapping

**Figure 4:**
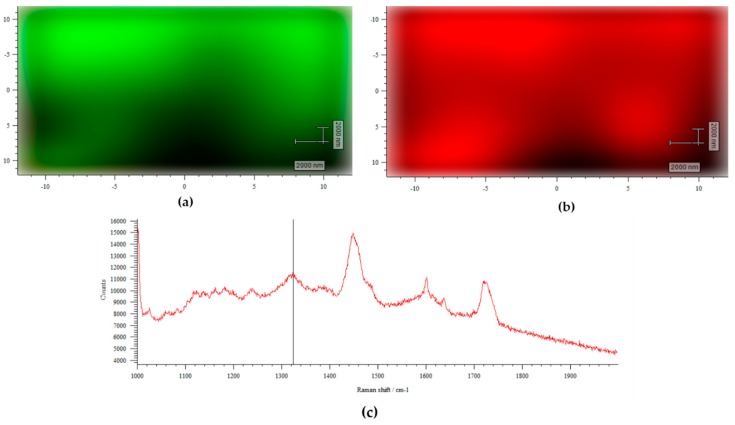
Raman surface mapping of ABC 0.3% GO. (**a**) Green corresponds to GO and black to ABC matrix; (**b**) red corresponds to ABC matrix and black to GO; and (**c**) Raman spectra to ABC 0.3% GO.

The distribution map of the GO obtained by Raman spectroscopy was used to evaluate its dispersion in the ABCs matrix. The regions used to identify the load and the matrix were determined from the characteristic bands of each component previously measured. In Figure 4a, the green color corresponds to the nanosheets of GO and the black color to the PMMA matrix, while in Figure 4b the red color corresponds to the matrix and the black color to the GO. In both figures, it is observed that the GO is well distributed within the ABCs matrix, which can be beneficial for mechanical reinforcement. Figure 4c shows the Raman spectrum obtained for the ABC sample, in which, 1324 cm^−1^ is indicated like the Raman shift used as a marker for the GO. Other authors have reported similar findings on GO dispersed in different matrices, such as inorganic silica particles [37] or polymeric substrates [38,39], where Raman spectroscopy showed a comparable surface pattern, concluding the homogeneous distribution and compatibility of GO across the respective matrices.

#### 2.2.3. Thermal Characterization

• TGA

**Figure 5:**
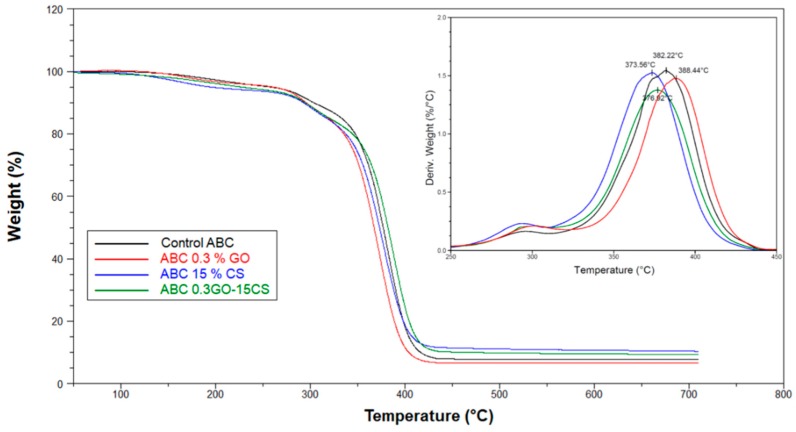
TGA and DTGA (inset) curves for experimental ABCs.

The TGA curves of the ABCs studied are shown in Figure 5. The thermal stability of the ABCs is reduced with the addition of GO or CS at temperatures below 400 °C. On the other hand, thermal stability increases with the addition of both fillers simultaneously, demonstrating a good dispersion of GO through the polymer matrix. The temperature, at which the maximum mass loss rate for formulations occurs, represented by the peaks of the graph of the first derivative of the TGA (DTGA), is increased by the presence of the GO and is decreased by the presence of the CS due to the individual thermal behavior of each component.

#### 2.2.4. Mechanical Characterization

• Compressive Strength

**Figure 6:**
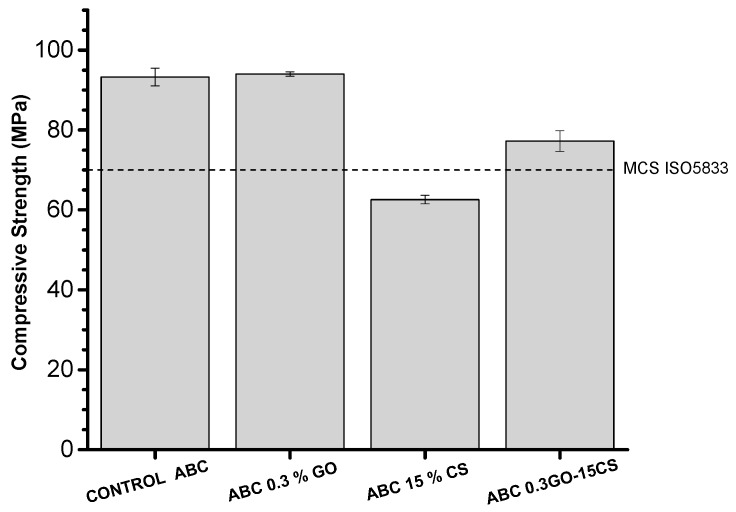
Compressive strength of the control ABC and experimental cements formulated with graphene oxide and chitosan. The dotted line indicates the minimum compressive strength (MCS) threshold defined by ISO5833.

Figure 6 shows that the compressive strength of the ABCs varies with composition. The incorporation of 15% of CS generates a considerable reduction of the resistance to values lower than the minimum compressive strength required in the MCS-ISO-5833 [40]. This might be due to the porosity generated by this component, the irregular shape of the sheets of CS that contain sharp edges that work as stress accumulators, and the lower amount of PMMA in the formulation. Moreover, it is possible that the presence of CS in the nanocomposite interferes with the polymerization of liquid monomer and, thus, causes significant decreases in the mechanical strength [41]. The addition of 0.3% GO to the ABCs does not present a significant difference in the compressive strength concerning the control formulation. However, when the GO is added to the formulation with 15% CS (ABC 0.3GO-15CS), the compression strength of this cement rises above the MCS ISO 5833 threshold.

The above is an indication that there is some hydrogen bonding interaction between the CS and the GO that favors the mechanical reinforcement in the cement and gives the cement a compression resistance superior to the MCS ISO 5833 threshold. Mechanical reinforcement of CS with GO has been widely reported in the literature [11,26] and is attributed to the homogeneous distribution of the GO and the interaction of the amino groups of the CS with the carboxyl groups of the surface of the GO through hydrogen bonds.

• DMA

**Figure 7:**
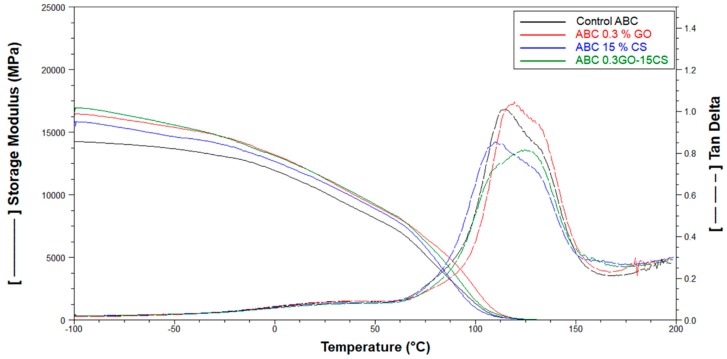
DMA results for the control ABC and experimental cements formulated with graphene oxide (GO) and chitosan (CS).

It is known that the behavior of the storage module determined by DMA, is related to the flexural modulus of the material. From results of this parameter for ABCs it can be inferred that the addition of the fillers improves the flexural behavior of the cements, reaching the highest increases in the two formulations containing GO. The glass transition temperature (T_g_) calculated from the tan δ (Table 1 and Figure 7), shows similar behavior to the compressive strength, finding a reduction in this parameter with the incorporation of CS mainly due to the lower degree of mechanical reinforcement that is related to the hardness of the material. Marques et al. [42] reported glass transition values of materials modified with biodegradable polymers similar to those found in this investigation. 

On the other hand, the content of GO increased the T_g_ thanks to the higher mechanical support provided by this component with nanometric size and the physical interactions between the polymer chains and the nanoparticles [43].

#### 2.2.5. Biological Characterization

• Hydrolytic Degradation

**Figure 8:**
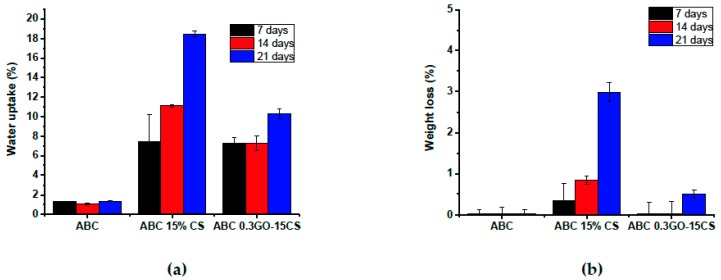
(**a**) Water uptake and (**b**) weight loss of experimental ABCs cements after immersion in PBS (pH 7.4) at 37 ºC for 7, 14, and 21 days.

Swelling properties are fundamental in the cell-biomaterial interactions [44]. Water absorption of PMMA ABCs is known to be very low due to their hydrophobic nature [45], which leads to the in vivo formation of a fibrous membrane around the cement. In this work, the hydrolytic behavior of the experimental ABCs will be crucial due to the incorporation of a biodegradable component which will influence the tissue interactions. From the results shown in Figure 8a, it can be observed that when the samples are immersed in Dulbecco’s phosphate buffered saline (PBS), the absorption of water increases with the immersion time. The formulation containing only CS, had the highest water uptake at 14 and 21 days, mainly due to the higher porosity of this nanocomposite, observed by SEM (Figure 3c) but also, to the hydrophilicity of CS [46]. The water absorption of the ABC 0.3GO–15CS was lower which can be attributed to the formation of hydrogen bonds between CS and GO. As far as degradation behavior is concerned, according to Figure 8b, the most significant weight loss of ABCs occurs in that modified only with CS because this is a bioresorbable polymer that is hydrolyzed in aqueous media [47]. Nevertheless, weight loss of any experimental cement was not very high, which can guarantee the maintenance of their mechanical properties.

• Cell Viability

**Figure 9:**
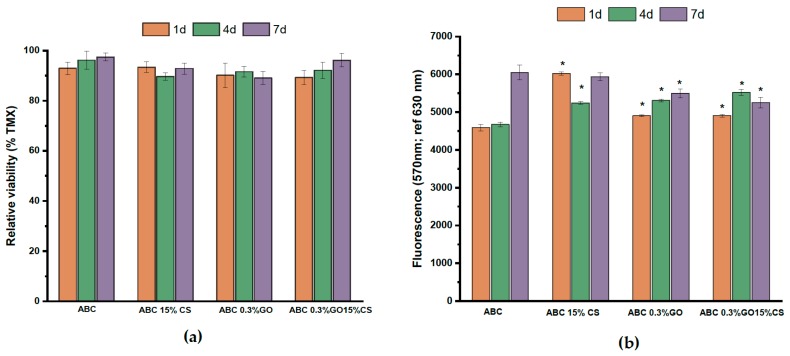
(**a**) Cell viability of human osteoblasts in the presence of extracts of ABCs taken after 1, 4, and 7 days; (**b**) Direct Alamar Blue results for control ABC and modified ABCs over a period of 1, 4, and 7 days. Results are the mean ± SD. Statistical analysis (*n* = 12) for each group was performed comparing experimental ABCs samples respect to ABC group at a significance level of *p* < 0.01.

Cytotoxicity of cements was evaluated with human osteoblasts cells (HOb) using the MTT assay that measures the succinate mitochondrial dehydrogenase enzyme activity [48]. According to Figure 9a none of the studied ABCs presented cytotoxicity for human osteoblasts since, in all cases, the cellular viability in the presence of extracts taken between one and seven days increases over time and is above 90%. Figure 9b shows the cell viability results when HOb are directly seeded on the ABCs surfaces. In this case, it can be seen that the presence of CS or GO in the cement formulation enhances the capacity of HOb for surface colonization and proliferation at early stages when compared with control ABC. This observation is in agreement with other authors that have reported the benefits of GO [49] and CS [50] for cell attachment and proliferation and indicates that all the studied formulations have a high potential to be used as a biomaterial in bone treatment applications.

• Osteogenic Properties

**Figure 10:**
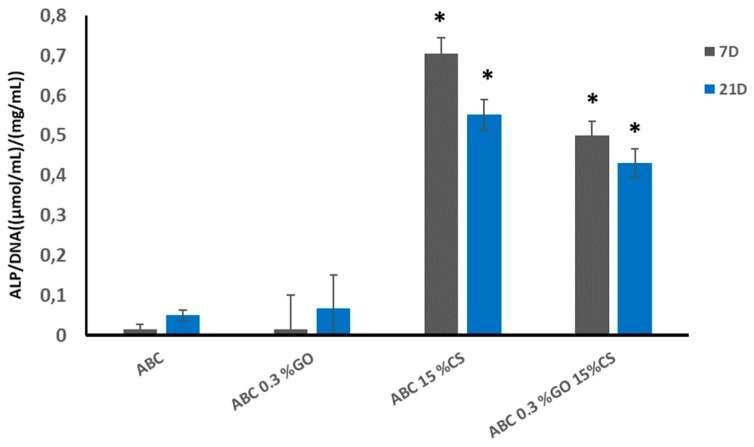
Variation of ALP activity expressed as the variation of p-nitrophenol concentration liberated in HOb cell cultures normalized by DNA content in the culture. Statistical analysis (*n* = 8) against control ABC group was performed at the significance level of *p* < 0.01.

Biochemical detection of ALP activity after seven and 21 days of incubation was used as an indicator of osteoblast phenotype [51]. Figure 10 shows that the modification of ABCs with chitosan clearly enhanced ALP activity from the early stages, thus inducing osteoblastic activity in both experimental ABC 15% CS and ABC 0.3GO-15CS groups. Although researchers Unagolla and Jayasuriya confirmed that the GO increased the activity of ALP PCL scaffolds [49], in this research we found that the greatest effect on the ALP activity was due to chitosan. This polysaccharide has been well studied for bone tissue engineering as osteoconductive and osteoinductive material [14], since it induces the proliferation of osteoblast and mesenchymal cells, and induces in vivo neovascularization [27]. Likewise, other researchers indicated that both GO and CS have a high potential in orthopedic tissue engineering [19] thanks to the fact that they have shown an increase in the proliferation of chondrocytes [21]. They induce attachment and proliferation of mouse mesenchymal stem cells (MSCs) and stimulate expression of the osteogenic gene osteocalcin, thus leading to differentiation of MSCs into bone [50]. They are cytofriendly to rat osteoprogenitor cells, enhance differentiation of MSCs into osteoblasts, and also accelerate bridging of the rat tibial bone defect with increased collagen deposition [52]. Additionally, they promote new bone formation on cranial defects of critical size in Wistar rats [53].

• Antibacterial Activity against *E. coli.*

**Figure 11:**
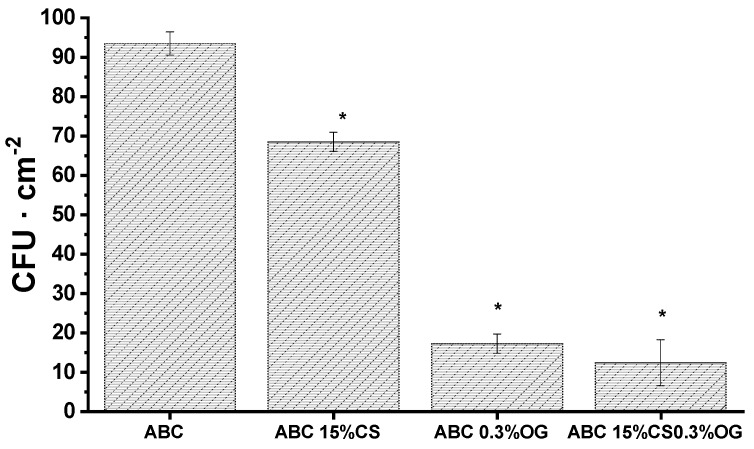
Number of colonies forming units recovered from ABCs surfaces after incubation of *E. coli* cultures for 24 h. Results are the mean ± SD. Statistical analysis (*n* = 12) for each group was performed comparing experimental ABCs samples respect to ABC group at a significance level of *p* < 0.01.

Immobilized CS and GO on ABC surfaces play a key role on the antibacterial effect of the experimental cements diminishing the number of viable colonies forming units on experimental cements as observed in Figure 11. However, it is clear that the main antibacterial capacity was observed in those ABCs formulated with GO. Raman surface analysis shown in Figure 4 confirmed the presence of GO sheets homogeneously distributed across the experimental ABC surfaces. Thus, this component would contribute mainly to the antibacterial activity as a result of cell membrane disruption caused by cell-surface interactions with GO [54].

As mentioned above, it is clear that the presence of GO in the formulations generated the highest antibacterial effect against *E. coli* bacteria. This is confirmed by the smaller colonies forming units present in the formulations that contained GO compared with the control cement and the ABC 15% CS after 24 h of incubation. On the other hand, the antibacterial effect of the ABCs generated by CS is lower than that provided by the GO, and the presence of both GO and CS in the cement produces a synergistic effect on its antibacterial activity. It is believed that the GO antimicrobial activity occurs when the sheets come into direct contact with bacterial cells, because of their negative charge and nano-laminar structure [55]. In this process, the cell membrane seems to be the main target of GO cytotoxicity. Membrane damage has been demonstrated in bacteria exposed to GO through morphological changes in the cellular structure, leakage of ribonucleic acid (RNA), electrolytes, intracellular absorption of membrane impermeable dyes, and changes in the transmembrane potential [56]. Additionally, oxidative stress is considered as an essential component of antimicrobial activity for bacterial cells exposed to GO [57].

## 3. Materials and Methods

### 3.1. Materials

Flake graphite (325 mesh, Alfa Aesar, Tewksbury, MA, USA), sulfuric acid ((H_2_SO_4_), potassium permanganate (KMnO_4_), hydrogen peroxide (H_2_O_2_), and 2-propanol (Merck, Burlington, MA, USA) were used for the synthesis of the GO. PMMA microparticles (New Stetic SA, Medellin, Colombia), barium sulfate (BaSO_4_,) (Alfa Aesar, Tewksbury, MA, USA), benzoyl peroxide (BPO), and chitosan from shrimp shells (M_w_ = 190.000–310.000 g/mol and a deacetylation degree of 88%, Sigma-Aldrich, Palo Alto, CA, USA) were used in the preparation of the solid phase of ABCs. Methyl methacrylate (MMA), 2-(diethylamino) ethyl acrylate (DEAEA), 2-(diethylamino) ethyl methacrylate (DEAEM) (Sigma-Aldrich, Palo Alto, CA, USA), and *N*,*N*-dimethyl *p*-toluidine (DMPT) (Merck, Burlington, MA, USA) were used in the liquid phase. All materials were used as received from the supplier, except for the BPO, which was recrystallized from methanol, and the CS, which was purified by dissolving in a 0.5 M solution of acetic acid, precipitation in a 2 M sodium hydroxide solution, and washing in water:ethanol solutions with decreasing concentrations (1:9, 3:7, 1:1, 2:1, 1:0, 1:0). Then it was frozen, lyophilized, and finally milled and passed through a 150 μm sieve.

### 3.2. Synthesis of GO

The GO synthesis was carried out using the modified Hummers method, following the methodology proposed by Mangadlao et al. with some modifications [58]. Briefly, 3 g of graphite were mixed for 10 min in a flat bottom flask, and then 3 g of KMnO_4_ were added to the mixture. The reaction remained in constant agitation all the time and every 24 h, 3 g of KMnO_4_ were added three more times. The reaction was stopped 24 h after the last addition of KMnO_4_ by taking a third of the mixture (approximately 134 mL) and mixing it with 300 mL of an ice water/Milli Q water mixture. After 10 min 2 mL of H_2_O_2_ were added and stirring was continued for 10 min. The mixture was transferred to 50 mL falcon tubes and centrifuged for 10 min at 4000 rpm. The supernatant was discarded, and the sediment was washed twice with Milli Q water followed by centrifugation. The purification was then carried out using 2-propanol until reaching a neutral pH. The solution was dialyzed for three days in isopropanol using SnakeSkin Dialysis Tubing, 3.5K MWCO (Thermo Scientific, Suwanee, GA, US). Finally, two new washes were made with Milli Q water, after which the graphite oxide was frozen at −40 °C for at least 24 h and lyophilized at −51 °C and 0.12 mBar pressure for 24 h in a Freezone freeze dryer 4.5 (Labconco, Kansas City, MO, US). 

### 3.3. Characterization of GO

The interplanar distance of the GO sheets was measured by X-ray diffractometry (XRD) in a PANalytical X’Pert PRO diffractometer (Malvern Panalytical, Jarman Way, Royston, UK), using Cu Kα1 radiation (1.540598 Å) and Kα2 (1.544426 Å), in a 2θ range between 5° and 40°. The Raman spectrum was measured using an Invia Raman Microscope with a wavelength laser of 514.5 nm (Renishaw, New Mills, Gloucestershire UK). The thickness of the nanosheets was determined by atomic force microscopy (AFM) in tapping mode using a Multimode AFM (Vecco Instruments, Santa Barbara, CA, USA) equipped with a Nanoscope Iva control system (software version 6.14r1). Silicon tapping probes (RTESP, Veeco) were used with a resonance frequency of ~300 kHz, and a scan rate of 0.4 Hz, 5 × 5, 2 × 2 μm^2^ AFM images were taken for each sample. Topography was examined by topographical AFM. Finally, the size of the particles was determined in a Zetasizer Nano ZS DLS (Malvern Panalytical, Jarman Way, Royston, UK).

### 3.4. Preparation of ABC

The composition of the ABCs nanocomposites is shown in Table 2. In the solid phase, BaSO_4_ was used as a radiopaque agent and BPO acted as an initiator of the MMA free radical polymerization reaction. In the liquid phase, methyl methacrylate was the main monomer, and 2-(diethylamino) ethyl acrylate (DEAEA) and 2-(diethylamino) ethyl methacrylate (DEAEM) were used as comonomers to improve the bioactivity of ABCs. The DMPT was the activating agent of the polymerization reaction because it decomposes the BPO through a process of oxide-reduction by transfer of electrons to initiate the polymerization reaction at room temperature. CS and GO were added as bioactive fillers.

The GO was dispersed in the liquid phase for 1 h by ultrasound (J.P. Selecta, Barcelona, Spain) and the cements were prepared manually by mixing the solid and the liquid, maintaining the solid/liquid ratio in 2 g/mL. The cement paste was placed into Teflon molds with the shape required for each test.

### 3.5. Characterization of ABC

#### 3.5.1. Chemical Characterization

• Infrared Spectrometry with Fourier Transform (FTIR)

Combined attenuated total internal reflectance/Fourier transform infrared (ATR-FTIR) spectroscopy spectra were recorded on a Spectrum One spectrometer equipped with an ATR accessory (Perkin-Elmer, Waltham, MA, US).

• Residual Monomer

Proton nuclear magnetic resonance spectra (^1^H NMR) were recorded in a Bruker Avance III HD-400 equipment at 25 °C. ABCs were dissolved in deuterated chloroform (CDCl_3_) one week after being prepared. The residual monomer (RM) was calculated by integrating the signals of the methoxyl protons of the MMA and the PMMA using Equation (1):

(1) $$RM\left(\%\right)=\frac{A_{MMA}}{A_{MMA}+A_{PMMA}}\times 100$$

where *A_MMA_* is the signal area of methoxyl protons of MMA (δ = 3.7 ppm), and *A_PMMA_* is the signal area of methoxyl protons of PMMA (δ = 3.5 ppm). A minimum of five replicates were analyzed per sample. Values were given as average ± standard deviation (SD).

#### 3.5.2. Physical Characterization

• Scanning Electron Microscopy (SEM)

The morphology and structure of ABCs were observed using an environmental scanning electron microscope (ESEM) Philips XL30 with tungsten filament (Philips, Eindhoven, The Netherlands). ABC cylinders with the dimensions of the compression specimens were cooled with liquid nitrogen and subsequently fractured. SEM examination was performed on the fracture zone.

• Raman Spectroscopy

Surface mapping was measured with an Invia Raman microscope (Renishaw, New Mills, Gloucestershire UK) using a 785 nm diode laser. The Raman band of GO about 1324 cm^−1^ and the band of ABC matrix about 1448 cm^−1^ were used as wavenumber markers. The Raman mapping of ABC with GO was done over 20 × 20 μm^2^ scanning areas.

#### 3.5.3. Thermal Characterization

• Thermogravimetric Analysis (TGA)

Thermogravimetric analysis (TGA) curves were obtained in a thermogravimetric TGA Q500 instrument (TA instruments, New Castle, DE, US). Samples were analyzed in a range of 50–500 °C under nitrogen at a heating rate of 10 °C/min. From the thermograms, the thermal stability was obtained.

#### 3.5.4. Mechanical Characterization

• Compressive Strength

The compression test specimens consisted of cylinders of 6 mm in diameter and 12 mm in height, and the cement was tested 24 h after mixing. The test was performed on an Instron 3366 universal testing machine (Instron, Norwood, MA, US) with a 5 kN cell at a load application speed of 20 mm/min according to ISO 5833 [40].

• Dynamic Mechanical Analysis (DMA)

The test was performed in a DMA Q800 (TA instruments, New Castle, DE, US), in dual cantilever mode. The frequency used was 1 Hz, with a working width of 20 μm. The temperature used was from −100 to 200 °C with a heating rate of 3 °C/min and an isotherm at −100 °C for 10 min. The specimens consisted of rectangular beams of 46 mm × 6 mm × 4 mm. The glass transition temperature and the storage modulus were determined from this test.

#### 3.5.5. Biological Characterization

• Hydrolytic Degradation

The hydrolytic degradation test was carried out following the specifications of ASTM F1635-16 [59]. Cylindrical test pieces 6 mm in diameter and 12 mm in height were immersed in Dulbecco’s phosphate buffered saline (PBS) (Sigma-Aldrich, St. Louis, MO, US) for 7, 14, and 21 days. The initial weight of the samples before immersion was recorded as *W*_0_, the wet weight after immersion and surface drying with the absorbent paper was recorded as *W_w_* and the weight after drying for at least 72 h in the incubator at 37 °C registered as *W_d_*. The weight loss (*W_t_*) and the water absorption were calculated by Equations (2) and (3), respectively. In all the experiments, a minimum of three samples were averaged:

(2) $$W_{t}\;\left(\%\right)=\frac{W_{0}-W_{d}}{W_{0}}\;\times 100$$

(3) $$\%\;water\;uptake=\frac{W_{w}-W_{d}}{W_{d}}\;\times 100$$

• Cell Viability

##### MTT Assay

Methylthiazol tetrazolium (MTT) test was used to determine the cell toxicity of the ABC in the presence of human osteoblasts cells (ECACC 06090739). Disc-shaped specimens of 10 mm in diameter and 1.5 mm thickness sterilized were submerged in 5 mL of complete medium (Dulbecco’s modified Eagle’s medium (DMEM)) and incubated at 37 °C for 1, 4, and 7 days. 

Human osteoblast (HOb) cells at the 8th passage were seeded in 96-well plates at a density of 9 × 10^4^ cell/well, and 100 µL of the corresponding exposure medium were added to each well. The complete medium was used as a target. The cytotoxicity of the ABC extracts was assessed by MTT (Sigma, UK) assay. The human osteoblast cells seeded in 96 well plates were exposed to the sample extracts for 24 h. The medium was then replaced by 100 µL of 0.5% MTT solution in phosphate buffered saline (PBS), and the cells were incubated at 37 °C for another 4 h. After that, the blue precipitated formazan dye was dissolved by the addition of 100 µL of dimethylsulphoxide (DMSO).

The optical density of the wells was measured at 570 nm using a microplate reader Synergy HT (Biotek, Winooski, VT, US). Percentage of cell viability was calculated by Equation (4):

(4) $$\%\;Cell\;viability=\frac{DO_{s}-DO_{T}}{DO_{C}}\;\times 100$$

where *DO_S_*, *DO_T_*, and *DO_C_* are the optical density measurements of the sample, the target (MEM introduced into wells without cells) and the negative control (Thermanox), respectively.

##### Alamar Blue Assay

HOb cells were seeded at a density of 9 × 10^4^ cell/mL and cultured for 24 h over the testing specimens placed in 24-well culture plate. After that, 2 mL of Alamar Blue dye (10% Alamar Blue solution in phenol red free DMEM medium) were added to each specimen. After 4 h of incubation 100 µL (*n* = 4) of culture medium for each test sample were transferred to a 96-well plate, and the absorbance was measured at 490 nm on a Biotek ELX808IU. The specimens were washed with PBS twice to remove the rest of the reagent, and 1 mL of culture medium was added to monitor the cells over the materials. This step was done at 1, 4, and 7 days. Results are given as mean ± SD. Analysis of variance (ANOVA) of the results for experimental ABCs was carried out with respect to control ABC at a significance level of *p* < 0.01.

• Osteogenic Properties

##### ALP Assay

The alkaline phosphatase (ALP) activity was evaluated using a colorimetric assay kit (Abcam, USA, ab83369) in confluent HOb cells seeded on the surface of sterilized disc-shaped specimens of 10 mm in diameter and 1.5 mm thickness (9 × 10^4^ cell/disc) after seven and 21 days of incubation. ALP catalyzes the hydrolysis of p-nitrophenyl phosphate (pNPP) to p-nitrophenol. It has a strong absorbance at 405 nm. The rate of the increased absorbance at 405 nm is proportional to the enzyme activity. Cell-free specimens were run alongside to correct for nonspecific background activity and all the results were normalized to DNA content as quantification for the number of cells. Total DNA was measured using the PicoGreen dsDNA quantitation kit (Madrid, Spain, Fisher Scientific, P-7589, Molecular Probes). Results are given as the mean ± SD. ANOVA of the results of tested materials was performed comparing the corresponding experimental ABC with respect to control ABC at each time point (*p* < 0.01).

• Antibacterial Activity against *E. coli*

Antibacterial activity of ABCs loaded with CS and GO was evaluated versus Gram-negative bacterium *E. coli* (DH5α). Bacterial concentration was estimated spectrophotometrically at 600 nm and adjusted with sterile PBS to an OD600 equivalent to 10^8^ CFU/mL. The ability of bacteria to form biofilms was analyzed by colony-forming units (CFU) determined by standardized plate counting agar techniques. Briefly, sample discs of 10 mm diameter were placed into a separate well of a 24-well plate with the test surface facing up. The plate was introduced in a plastic container wet filter paper beneath to maintain a relative humidity of 90%. 100 µL of test inoculums (10^5^ CFU·cm^−2^) prepared in 1/500 diluted Nuria Broth medium (Sparks, MD, USA, Difco) were placed onto the substrates and incubated for 24 h at 37 °C. Afterwards, samples were washed with 1 mL of PBS, (pH 7.2) and the numbers of CFU recovered from each sample disks were determined by standardized plate counting agar techniques and referred as CFU (N)/cm^2^. The mean reported for each concentration of filler was based on three replicates.

### 3.6. Statistical Analysis

Statistical analysis was performed using a Student’s t-test. Values of compressive strength, degradation, cell viability and antibacterial activity were presented as the mean ± SD. Differences were considered statistically significant at *p* < 0.01.

## 4. Conclusions

The results of this research showed that all the ABCs developed formulations presented excellent biocompatibility, which shows their potential to be used in biomedical applications. Moreover, the presence of GO in the ABCs provided mechanical reinforcement and antimicrobial activity while the addition of CS favored the generation of pores in the cement, as evidenced by SEM, decreased the compression strength and increased the bioactivity of the cement, increasing the ALP activity. 

A synergistic effect on the physical, thermal, mechanical, and antibacterial properties, along with a rise in the osteogenic activity was evidenced for the ABC nanocomposites formulated with both 15% of CS and 0.3% of GO (CS + GO). These results suggest that this formulation possesses a high potential to be used as an antibacterial bioactive cement in orthopedic applications.

## Figures and Tables

**Table 1:** Residual monomer and glass transition temperature of ABCs.

Formulation	Residual Monomer (%)	T_g_ by DMA (°C)
Control ABC	1.1	114.8
ABC 0.3 % GO	1.3	119.6
ABC 15 % CS	1.6	109.5
ABC 0.3GO-15CS	3.6	124.5

**Table 2:** Composition of ABCs formulations.

Formulation	Solid Phase (% *w/w*)				Liquid Phase (% *w/w*)			
	PMMA	BaSO_4_	BPO	CS	MMA	DEAEM/ DEAEA	DMPT	GO
Control ABC	88	10	2	0	95.5	2	2.5	0
ABC 0.3 % GO	88	10	2	0	95.2	2	2.5	0.3
ABC 15 % CS	73	10	2	15	95.5	2	2.5	0
ABC 0.3GO-15CS	73	10	2	15	95.2	2	2.5	0.3

## Supplementary Materials

Supplementary materials can be found at https://www.mdpi.com/1422-0067/20/12/2938/s1.
