# Supplementary material for: Novel Bioactive and Antibacterial Acrylic Bone Cement Nanocomposites Modified with Graphene Oxide and Chitosan
Source: Int J Mol Sci. 2019 Jun 15;20(12):2938. doi: 10.3390/ijms20122938 (PMC6627441; doi:10.3390/ijms20122938)
Supplement: Supplementary file 1 [file ijms-20-02938-s001.pdf]

International Journal of *Molecular Sciences*

MDPI

Article

Novel Bioactive and Antibacterial Acrylic Bone Cement Nanocomposites Modified with Graphene Oxide and Chitosan

Mayra Eliana Valencia Zapata [1], José Herminsul Mina Hernandez [1], Carlos David Grande Tovar [2,*], Carlos Humberto Valencia Llano [3], José Alfredo Diaz Escobar [4], Blanca Vázquez-Lasa [5,6], Julio San Román [5,6] and Luis Rojo [5,6,*]

[1] Grupo de Materiales Compuestos, Escuela de Ingeniería de Materiales, Universidad del Valle, Calle 13 # 100-00, Cali, Colombia; valencia.mayra@correounivalle.edu.co (M.E.V.Z.); jose.mina@correounivalle.edu.co (J.H.M.H.)

[2] Grupo de Investigación de Fotoquímica y Fotobiología, Universidad del Atlántico, Carrera 30 Número 8-49 Puerto Colombia, Colombia;

[3] Grupo Biomateriales Dentales, Escuela de Odontología, Universidad del Valle, Calle 13 No. 100-00, Cali, Colombia; carlos.humberto.valencia@correounivalle.edu.co

[4] Departamento de Ciencias Básicas, Institución Universitaria Antonio José Camacho, Avenida 6N # 28N – 102, Cali, Colombia; afro77777@gmail.com

[5] Instituto de Ciencia y Tecnología de Polímeros, ICTP-CSIC, C/Juan de la Cierva 3, 28006 Madrid, Spain; bvazquez@ictp.csic.es (B.V.-L.); jsroman@ictp.csic.es (J.S.R.);

[6] Consorcio Centro de Investigación Biomedica en red, CIBER-BBN, 28029, Madrid, Spain.

* Correspondence: carlosgrande@mail.uniatlantico.edu.co (C.D.G.T.), rojodelolmo@ictp.csic.es (L.R.);

Received: 3 May 2019; Accepted: 12 June 2019; Published: date

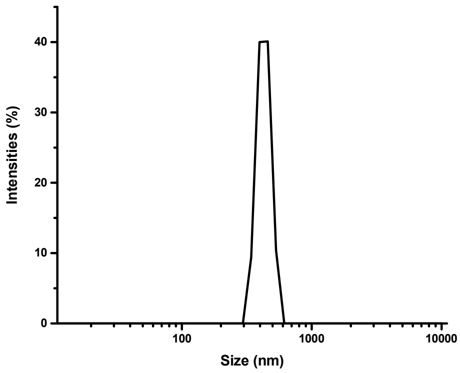

Figure 1. Particle size by Dynamic light scattering (DLS).
